# Identifying Risk Profiles of School Refusal Behavior: Differences in Social Anxiety and Family Functioning Among Spanish Adolescents

**DOI:** 10.3390/ijerph16193731

**Published:** 2019-10-03

**Authors:** Carolina Gonzálvez, Ángela Díaz-Herrero, Ricardo Sanmartín, María Vicent, Antonio M. Pérez-Sánchez, José M. García-Fernández

**Affiliations:** 1 Department of Development Psychology and Teaching, Faculty of Education, University of Alicante, 03690 Alicante, Spain; carolina.gonzalvez@ua.es (C.G.); ricardo.sanmartin@ua.es (R.S.); maria.vicent@ua.es (M.V.); am.perez@ua.es (A.M.P.-S.); josemagf@ua.es (J.M.G.-F.); 2 Department of Development Psychology and Education, Faculty of Psychology, University of Murcia, 30003 Murcia, Spain

**Keywords:** school refusal behavior, social anxiety, family functioning, adolescents, latent class analysis

## Abstract

School attendance problems negatively affect students’ development. This study attempted to identify different school refusal behavior profiles and to examine their relationship with three dimensions of social anxiety (fear of negative evaluation, social avoidance and distress in new situations, and social avoidance and distress that is experienced more generally in the company of peers) and the perception of family functioning. Participants included 1842 Spanish adolescents (53% girls) aged 15–18 years (*M* = 16.43; *SD* = 1.05). The School Refusal Assessment Scale—Revised (SRAS-R), the Social Anxiety Scale for Adolescents (SAS-A), and the Family APGAR Scale (APGAR: Adaptation, Partnership, Growth, Affection, and Resolve) were administered. Latent class analysis revealed four school refusal behavior profiles: non-school refusal behavior, high school refusal behavior, moderately low school refusal behavior, and moderately high school refusal behavior. Analyses of variance (ANOVA) indicated that adolescents’ with the profile of high school refusal behavior showed higher scores in all the subscales of social anxiety. In contrast, the non-school refusal behavior group revealed higher scores in the perception of good family functioning, whereas the high school refusal behavior profile obtained the lowest scores in this scale. These findings suggest that students who reject school are at a higher risk of developing social anxiety problems and manifesting family conflicts. These students should be prioritized in order to attend to their needs, promoting self-help to overcome social anxiety and family problems with the purpose of preventing school refusal behaviors.

## 1. Introduction

School refusal behavior (SRB) is understood as a broad term that includes students who refuse to attend school or show persistent difficulties in remaining in class that may or may not be behaviors based on anxiety [1]. Pina et al. [2] pointed out that up to 28% of students express SRB at any time throughout their academic career. In addition to the high incidence rates, SRB is considered a major problem associated with numerous negative consequences for young people, such as behavioral disorders [3]; lower academic achievement [4,5]; significant psychopathology; most commonly, depression and anxiety [6]; involvement in pre-criminal behavior and the consumption of substances such as alcohol and drugs [7]; and, on occasion, even school dropout [8]. In terms of the public health impact, these findings warn about the association between school absenteeism and risk behaviors, including alcohol use, tobacco use, other drug use, and risky sexual behaviors [9]. The association of school absenteeism with these kinds of health problems has been documented since the 1970s [10] and has persisted into the last decade [11,12].

Traditionally, clinicians and researchers have grouped children who show school attendance problems into two major categories: those who remain at home instead of attending school because of fear or anxiety and those who do not attend classes due to the lack of interest or challenging behavior [13]. However, according to Kearney [14], this classification is too generic and does not accurately describe the totality of this population, and it is not linked to effective evaluation and treatment strategies. In order to determine the different types of school attendance problems, two contemporary approaches have been developed. On the one hand, Heyne et al. [15] proposed distinguishing between four types of school attendance problems named school refusal, truancy, school withdrawal, and school exclusion, measured by the School Non-Attendance Checklist (SNACK). On the other hand, Kearney and Silverman [16] proposed a functional analytic model based on the principles of reinforcement theory. This model comprises an assessment of negative and positive reinforcing behaviors through four functional factors that describe why a child is not attending school. In this study, SRB was assessed according to the four functional factors modeled via the School Refusal Assessment Scale (SRAS) [17]. This instrument has been widely internationally recognized, as well as its revised version, the School Refusal Assessment Scale—Revised (SRAS-R) [18], which has shown adequate psychometric properties in Spanish-speaking populations [19,20,21]. According to this approach, there are four functions that underlie SRB, as follows: (1) avoidance of school-based stimuli that provoke negative affectivity, (2) escape from aversive social and/or evaluative situations, (3) pursuit of attention from significant others, and/or (4) pursuit of tangible reinforcements outside of school. The first two dimensions refer to school refusal behavior maintained by negative reinforcement and the last two dimensions refer to that maintained by positive reinforcement.

### 1.1. School Refusal Behavior Profiles

Based on the functional analytic model, it would be expected that more than one dimension or factor could be presented as generators of SRB within the same group of individuals [17]. Consequently, identifying profiles of students that share similar school refusal characteristics could allow us to offer prevention and intervention strategies in line with the needs of each subgroup. In this sense, Berg et al. [22] and Bools et al. [23] established four groups with non-clinical samples of children and adolescents (*N* = 80; *N* = 100, respectively): an absentee group (absent without parental knowledge), a group of school refusal (related to anxiety), a mixed group (including characteristics of the previous two groups), and a group without school refusal. To do this, they used parents’ interviews as instruments to assess children with this problem. Later, Dube and Orpinas [24] analyzed the SRB profiles in a non-clinical sample of 99 North American students (*M* = 12.5, *SD* = 1.38, range = 8–15 years) with school attendance problems. In this study, based on the average scores on the dimensions of the School Refusal Assessment Scale for Children (SRAS-C), three profiles were identified: a mixed reinforcements profile of school refusal that combined explanatory factors for both positive and negative reinforcement; a positive reinforcement profile of school refusal, which included factors related to obtaining attention from significant persons or seeking tangible reinforcements outside the school; and a profile of non-school refusal.

In recent years, the interest in knowing the different profiles of students who reject school has reached Spanish-speaking countries. On the one hand, in Spain, Gonzálvez et al. [25,26] identified four SRB profiles using a multistage random cluster sampling methodology in a non-clinical sample of 1113 Spanish children aged 8–11 years (*M* = 9.53; *SD* = 1.10). The four groups identified were called non-school refusers, school refusers by positive reinforcements, school refusers by negative reinforcements, and school refusers by mixed reinforcements. Later, these profiles were corroborated by a study carried out with a community sample of 1212 Spanish children in the same age range (*M* = 9.12, *SD* = 1.05) using cluster analysis [27]. On the other hand, in Ecuador, only the study carried out by Gonzálvez et al. [28] identified three SRB profiles in a non-clinical sample of 1582 adolescents (*M* = 14.83, *SD* = 1.86). In this case, the SRBs identified were non-school refusers, school refusers by tangible reinforcements, and school refusers by mixed reinforcements.

### 1.2. School Refusal Behavior and Social Anxiety

School refusal behavior profile studies not only distinguish between groups of students with school attendance problems but also analyze the relationship of these profiles with other variables fundamentally related to internalizing problems (e.g., anxiety, depression, stress). This is because anxiety disorders have high rates of incidence and prevalence in this population, and their effects can be detrimental to human development in all stages of life [29]. However, during adolescence, problems in emotional control and social relationships can adversely affect the proper adjustment of young people. This is because adolescence is considered one of the most vulnerable stages of the life cycle, after childhood, due to the changes that occur at a biological, social, and psychological level [30]. Ensuring the healthy development of young people and adolescents is a basic element for the social, economic, and political advancement of any country.

In adolescence, social anxiety disorder is associated with negative repercussions for academic and social development. Regarding the academic field, several studies have found that adolescents who suffer from this disorder refuse to participate in class, engage in public presentations of work, or ask the teacher questions in both public and private [31]. These behaviors generally cause students to obtain marks below their abilities; present higher rates of school absenteeism [32]; and in many cases, even drop out from their academic studies [33]. Likewise, adolescents with high social anxiety have fewer friends, which leads to them experiencing more feelings of loneliness [31,34] and to present lower levels of acceptance and support from their peer group, leading them to be more susceptible to being ignored, rejected, and ridiculed [35,36,37]. These facts are of special relevance in this phase of life since it is a critical moment for the development of feelings of personal competence, socialization, and the development of relationships and interpersonal skills [38,39,40].

Previous studies have shown that anxiety disorders, and specifically social anxiety, show comorbidity with SRB. In a retrospective study [33], it was found that almost half of a sample of adults with anxiety disorders reported having left school prematurely. The reasons that were given indicated that 22.4% experienced anxiety (feeling nervous in class or at school) and 16.9% showed behaviors related to anxiety (problems participating in class). The results of a more recent investigation carried out by Richards and Hadwin [41], with a community sample of 162 adolescents between 12 and 13 years of age, also revealed negative relationships between trait anxiety and school attendance that were mediated by the factors of avoidance of social situations and searching for the attention of significant persons postulated by Kearney and Silverman [17]. Specifically, these data indicated that high anxiety was linked to a tendency to avoid social situations and a growing desire to spend more time with other significant people. In the same line, Kearney and Albano [42] administered the School Refusal Assessment Scale (SRAS) [17] in a clinical sample of 143 children from 5 to 17 years of age and their parents. The results obtained indicated, on the one hand, that diagnoses related to anxiety were more associated with negatively reinforced school refusal behavior and, on the other hand, that separation anxiety disorder was more related to seeking attention from significant people. In contrast, behavioral disorders and defiant negative disorder were more associated with the search for tangible reinforcements outside of school.

Some of these studies established groups taking into account the presence or absence of anxiety in this behavior to analyze the consequences of SRB. In this line, Egger et al. [43], in a longitudinal investigation carried out with 1422 American children and adolescents aged between 9 and 16 years, found that the profile of school refusal based on anxiety was more associated with depression, separation anxiety disorder, and greater problems in relationships with peers, while the profile of truancy (without anxiety) was more related to behavioral disorders, oppositional defiant disorder, and depression. This study also examined a third group that showed characteristics of the two previous groups (children with anxious school refusal and truancy), and the results revealed that 88.2% of the subjects presented some psychiatric disorder with the presence of emotional and behavioral problems. On the other hand, Ingul and Nordahl [44] studied in a sample of 865 Norwegian students, whose ages ranged between 16 and 21 years (*M* = 17.21, *SD* = 1.28), the differences between groups with and without anxiety. The Screen for Child Anxiety-Related Emotional Disorders (SCARED) [45] was used to assess anxiety’s symptoms. Additionally, other variables such as depression, personality, resilience, externalizing behavior problems, substance use, and other contextual and sociodemographic factors were also assessed. The results indicated a greater psychiatric severity and behavioral problems related with anxiety in young people with a mixed profile, characterized by high levels of anxiety and truancy. As a result, this group scored higher on social anxiety and panic disorders.

These findings are consistent with those of other previous studies, such as those made by Bools et al. [23], Berg et al. [22], and Dube and Orpinas [24]. These studies also found that disruptive behavior disorders were associated with truancy, while anxiety and mood disorders were linked to anxiety-based school refusal [22,23]. It was also noted that children and adolescents belonging to the profile that combined anxious school refusal and truancy showed both behavioral and emotional problems [23,24]. In the same way, in the works with Spanish and Ecuadorian samples, the profiles of school refusal by mixed reinforcement were the most maladaptive, obtaining higher scores in anxiety, depression, or stress [25,26,28].

### 1.3. School Refusal Behavior and Family Functioning

In recent years, research on problems that affect adolescents has undergone a major paradigm shift from an approach focused on the analysis of individual development to the influence of social contexts [46]. At an individual level, the theoretical review shows that numerous studies have investigated the comorbidity of school refusal behavior with other internalizing problems (e.g., anxiety, depression, stress) [33,41,42]. However, in line with the new approach, the family context has received special attention due to its unquestionable importance as a basic social unit responsible for facilitating and protecting the growth and learning processes of its descendants. The family is the place where the adolescent develops from birth, so his characteristics will affect his adjustment and his life satisfaction. Although in the last decades there have been changes in the conceptualization and structure of families, the family environment is still a system that gives its members a family identity, as well as the transmission of values and patterns of behavior to effectively participate in life [47,48]. In this sense, the study of the perception of family functioning has gained great interest in the field of health and psychology in recent years. Family functioning is understood as the perception of the care and support that a person receives from their own family [49].

During adolescence, young people undergo rapid changes in terms of physical aspects and constant personal, social, family, and academic readjustments. In addition, they face the construction of their own identity from a particularly critical perspective with the different contexts in which they live, especially the family. Although family conflicts are an inevitable part of living together, problems in family relationships can increase during adolescence [50]. In this regard, it should be noted that problematic family functioning has been associated with school refusal in children and adolescents [51,52].

Several research studies have noted that families of youth with school attendance problems are less likely to be well functioning [53]. In fact, families characterized by poor cohesion and considerable conflicts or isolation are associated with youth with SRB [54,55]. Bernstein et al. [56] found family difficulties on the Family Assessment Measure (FAM; [57]) in the areas of role performance and values and norms in families of children with school refusal. “Role performance” assesses role definition and family members’ adaptation to new roles throughout the life cycle, whereas “values and norms” measures the degree of concordance among components of the family’s value system and the degree of agreement with the values of the culture in which the family lives. A few years later, using this measure and distinguishing between family constellations (single-mother family versus intact family), Bernstein and Borchardt [58] evaluated 134 families of children with school refusal. Single-parent families reported significantly more family problems in the areas of role performance and communication subscales than families with two biological parents. The “communication” area evaluates mutual understanding and the ability to seek clarification in case of misunderstanding.

Considering the familial relationship subtypes of children and adolescents with school refusal behavior (e.g., the enmeshed family, the conflictive family, the isolated family, the detached family, and the healthy family, as well as mixed familial profiles), Kearney and Silverman [59] evaluated 64 families using the Family Environment Scale (FES) [60]. The results revealed higher scores in the cohesion and expressiveness subscales in healthy families’ profiles, whereas the enmeshed family subtype scored worse on the independence subscale and the conflictive family profile obtained higher scores on the conflict subscale. On the other hand, distinguishing between students who attend school regularly (*N* = 27) and youth with school attendance problems (*N* = 27), Smith et al. [61] assessed the influence of family factors across the Children’s Report of Parental Behavior Inventory (CRPBI—Revised; [62]). The results revealed that absenting students scored lower on perceived cohesion in the family, parental acceptance, and parental discipline, whereas they scored higher on perceived conflict in the family.

Focusing on school refusers with comorbid anxiety disorders and major depression, Bernstein et al. [63] administered the Family Adaptability and Cohesion Evaluation Scale II (FACES II; [64]) to 46 adolescents and to their parents. Low cohesion and low adaptability were reported by the participants. In support of these findings, most recent scientific evidence affirms that unhealthy family relationships (e.g., overprotectiveness, ineffective parental control, separation and divorce, dysfunctional family interactions, parental psychopathology, poverty) have also been associated with school absenteeism [65], as well as with parent mental health [66] 

These studies highlight several patterns of problematic family functioning associated with school attendance problems. However, there is no previous research that has analyzed the perception of family functioning based on each profile or group of students who reject school. Latent class analysis is currently considered one of the best estimation methods to categorize subjects compared to other statistical tools such as cluster analysis [67]. Having these analyses to determine the different groups of youths with school attendance problems and identify, depending on the group they belong to, their perception regarding family functioning would not only have a descriptive impact but also help in the development of preventive measures and intervention [68].

### 1.4. The Present Study

The review of the literature indicates that anxiety symptoms and anxiety disorders, especially social anxiety, are associated with SRB [42,44,69,70], and the family environment is an important contextual risk factor for school non-attendance [54,55]. However, many of these investigations have been carried out with clinical and small populations, despite SRB being a frequent problem in the school environment. In addition, no previous SRB profiles have been identified for Spanish adolescents. Hence, the present study addresses this issue in a community sample of Spanish adolescents and aims to provide new empirical evidence on the relationship between SRB and social anxiety using the Social Anxiety Scale for Adolescents (SAS-A) [39] and family functioning through the Family Adaptation, Partnership, Growth, Affection, and Resolve (APGAR) Scale [49], neither of which have been previously used in this field.

On the other hand, although it is true that some studies have established SRB behavior profiles based on the functional assessment of this behavior [21,24,25,26], there are a lack of investigations that have contrasted the profiles identified with Spanish adolescents using latent class analysis, with no subset of structural equation modeling used to determine subtypes of groups more sophisticated than K-means clustering having been considered [67]. In turn, this is the first-ever study to disclose the effects of belonging to one group of students who reject school or another regarding social anxiety and perception of family functioning scores.

Based on these considerations, three aims are proposed: firstly, to identify the profiles of SRB in a community sample of Spanish adolescents, based on the four functional conditions of SRB established by the SRAS-R (first aim); and secondly, once the SRB profiles are found and defined, to analyze the existence of statistically significant differences in the social anxiety (second aim) and in family functioning scores depending on the different profiles identified (third aim). The hypotheses of the study are as follows: (1) It is expected that this study will identify four different groups or profiles of students with SRB, in line with the functional model proposed by Kearney and Silverman [17] and the results of Gonzálvez et al. [25,26], who found in Spanish children a group of non-school refusers, school refusers by positive reinforcements, school refusers by negative reinforcements, and school refusers by mixed reinforcements. (2) The profile of school refusal by multiple reinforcements (positive and negative reinforcements) is expected to be statistically significantly associated with higher scores in social anxiety than the rest of the groups [25,26,28,44]. (3) The group of school refusal by multiple reinforcements is expected to be the profile with a worse perception of family functioning [54,55,61].

## 2. Materials and Methods

### 2.1. Participants

A non-clinical sample was recruited by random sampling in 12 secondary education centers (north, south, east, west, and center) of Alicante and Murcia (Spain). In the participant selection process, only the data reported by students without any type of clinical diagnosis were selected. This information was provided by the school psychological services before filling in the questionnaires. Of the initial sample composed of 2021 students, 134 were excluded because they did not have written informed consent from their parents and 45 were excluded due to errors or omissions in completing the questionnaires. The final sample consisted of a total of 1842 students (53% girls) whose ages ranged between 15 and 18 years (*M* = 16.43, *SD* = 1.05) (see Table 1). The chi-square homogeneity test in the frequency distribution revealed the absence of statistically significant differences between the sex and age groups (χ^2^ = 3.12; *p* = 0.39).

The majority of students came from urban areas (83.2%). Regarding the socioeconomic level of the families, 25% had a medium–low level, 61% a medium level, and 14% a medium–high level, determined from family income and parents’ education level.

### 2.2. Measures

School Refusal Assessment Scale—Revised (SRAS-R) [18]: To measure SRB, the Spanish version of the SRAS-R was administered [28]. This is a self-reporting measure that measures SRB in children and adolescents and consists of 18 items that are answered on a 7-point Likert scale, ranging from 0 (never) to 6 (always). This instrument is composed of four factors that evaluate motivating conditions of school refusal behavior: avoidance of school-based stimuli that provoke negative affectivity (ANA; e.g., “How many times do you try not to go to school because if you go you will feel sad or depressed?”); escape from aversive social and/or evaluative situations (ESE; e.g., “If it were easier for you to make new friends, would it be easier for you to go to school?”); pursuit of attention from significant others (PA; e.g., “How many times would you prefer your parents to teach you at home instead of your teacher at school?”); and pursuit of tangible reinforcements outside of school (PTR; e.g., “How many times do you refuse to go to school because you want to have fun outside of school?”). High scores in one or more factors are indicators of greater school refusal and are considered as the main causes of its maintenance. The coefficients of internal consistency of this measure in this study were 0.74, 0.72, 0.81, and 0.71, respectively, for the four factors of the SRAS-R.

Social Anxiety Scale for Adolescents (SAS-A) [39]: This scale evaluates the experiences of social anxiety and fear of negative evaluation of adolescents in the context of relationships with peers. It consists of 22 items (4 of these are neutral and are not considered for obtaining the scores) that are evaluated on a Likert scale of 5 points that ranges from 1 (not at all) to 5 (all the time). It is composed of three subscales: fear of negative evaluation (FNE; e.g., “I worry about what others say about me”); social avoidance and distress in new situations (SAD-N; e.g., “I get nervous when I talk to peers I don’t know very well”); and social avoidance and distress that is experienced more generally in the company of peers (SAD-G; e.g., “I’m quiet when I’m with a group of people”). In this study we used the Spanish version of the SAS-A which has been shown to have adequate psychometric properties in the adolescent population [71], similar to those of the original version. The coefficients of internal consistency of this measure in this study were 0.76, 0.78, and 0.74, respectively, for the three factors of the SAS-A.

Family APGAR Scale (APGAR) [49]: This scale is a self-reporting measure that assesses the perception of family functioning through an exploration of the satisfaction degree in the relations that people have with their relatives. This scale consists of five items that are answered on a 5-point Likert scale, ranging from 0 (never) to 4 (always). Each item measures one of five components, respectively, (1) adaptation (family’s resources available for dealing with problems; e.g., “I am satisfied with the support I receive from my family when something concerns me”); (2) partnership (sharing of problems and decision-making by the family; e.g., “I am satisfied with how my family discusses issues of common interest and shares the problem solution with me”); (3) growth (the support for and acceptance of change in individual family members; e.g., “My family accepts my desires to promote new activities or make changes in my lifestyle”); (4) affection (the response of family members to the expression of feelings; e.g., “I am satisfied with how my family expresses affection and responds to my feelings of love and sadness”); and (5) resolve (satisfaction with the quality of time that family members spend together; e.g., “I am satisfied with the time my family and I share”). Satisfactory internal consistency reliability (α = 0.80) and test–retest reliability (*r* = 0.83) have been reported by previous research [57]. Similar results were obtained when translating this measure to Spanish for adolescents and an adult population (α = 0.84 internal consistency reliability; *r* = 0.75 test–retest reliability) [72]. The coefficient of internal consistency of this measure in this study was 0.82.

### 2.3. Procedure

During the initial visit to the different centers, the objectives of the research were explained and collaboration was requested. A letter was sent to the parents of the participants to inform them about the research and to request their written consent to participate in the study. The students completed the questionnaires voluntarily and anonymously during school hours, always under the supervision of a member of the research team. The average administration times were 20 min (SRAS-R), 10 min (SAS-A), and 10 min (APGAR). All procedures were performed according to the ethical standards of the 1964 Helsinki Declaration, and the research study protocol was approved by the ethical committee of the University of Alicante (code of ethics: UA-2017-09-05).

### 2.4. Statistical Analyses

First, to identify the SRB profiles, latent class analysis (LCA) was performed. The fit indices and the criteria taken into account to choose the most adequate class solution were the lowest Bayesian information criterion (BIC) and entropy values closer to 1 [67,73]. LCA advancements have provided a powerful technique to identify homogeneous classes of subjects in comparison with previously used statistical techniques, such as K-means for clustering or quartile clustering. The ability to use a wide variety of data with different variances along with regression and factor analysis options makes LCA an appealing option that quite easily overcomes all of the problems produced with K-means clustering [67]. The different profiles are the result of combining high, low, and moderate scores of the four factors of the SRAS-R (ANA, ESE, PA, and PTR).

Second, an analysis of variance (ANOVA) was conducted to examine the differences in the social anxiety subscales (FNE, SAD-N, and SAD-G) between the SRB profiles identified. To analyze the magnitude or size of the effect of these differences, the partial eta-squared index was taken into account (η^2^_p_), and post hoc tests (Bonferroni’s method) were performed to identify between which groups there were statistically significant differences. Likewise, the effect size was calculated (index d) [74] to obtain the magnitude of the differences observed. The index d is interpreted as follows: values between 0.20 and 0.49 indicate a low effect size, between 0.50 and 0.79 a moderate effect size, and above 0.80 a high effect size. The data were analyzed using the statistical package SPSS v22 and the Excel package (XLSTAT) to run the latent class analyses.

## 3. Results

### 3.1. Identification of School Refusal Behavior Profiles

**Figure 1:**
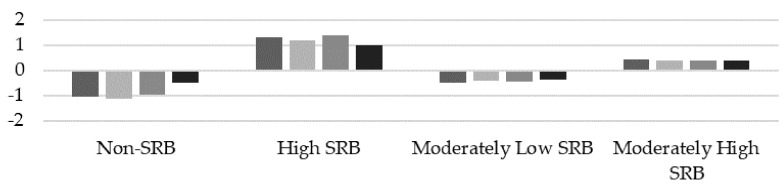
School refusal behavior (SRB) profiles.

According to the fit indices of the latent class analysis, the four-class model was the best fitting, having the lowest BIC and the highest entropy (see Table 2). Latent class analyses differentiated four groups of adolescents with SRB (see Figure 1), which was a result of the different combinations of dimensions of the SRAS-R (ANA, ESE, PA, and PTR). The first group consisted of 239 adolescents (12.9%) characterized by low scores for the four dimensions of the scale (ANA, ESE, PA, and PTR). This group was called non-school refusal behavior (Non-SRB). The second group included 251 adolescents (13.6%) who obtained high scores for the four dimensions (ANA, ESE, PA, and PTR). This group was called high school refusal behavior (High SRB), considered a mixed profile for combining high scores for both negative and positive reinforcement. The third group, called moderately low school refusal behavior (Moderately Low SRB), was formed of 741 adolescents (40.3%) and was defined by a moderately low score for the four factors of the SRAS-R. Finally, the fourth group, called moderately high school refusal behavior (Moderately High SRB), was formed of 611 adolescents (33.17%) and was characterized by moderately high scores for the four factors of the scale.

### 3.2. Intergroup Differences in Social Anxiety

Comparisons of the mean scores of each group in the three dimensions of the SAS-A and the Family APGAR Scale showed significant differences in all cases (see Table 3). The results showed that the High SRB group obtained the highest scores in all dimensions of the SAS-A, followed by the Moderately High SRB profile. In contrast, the lowest scores were obtained by the groups of Non-SRB and Moderately Low SRB. Regarding the Family APGAR Scale, for which high mean scores indicate a better perception of family functioning, the Non-SRB profile obtained the highest score for family function satisfaction, whereas the High SRB group exhibited the lowest score for this measure.

Table 4 shows post hoc comparisons revealing that the High and Moderately High SRB groups scored significantly higher than the Non-SRB group in all dimensions of the SAS-A, with large and moderate effect sizes (d between 0.69 and 0.88). Likewise, the High SRB group showed significantly higher scores in all the dimensions of the SAS-A in comparison with the group of Moderately Low SRB, obtaining moderate effect sizes in the first two dimensions (d = 0.60 and 0.58, respectively) and a low value for the third dimension (d = 0.48). Likewise, the group of Moderately High SRB scored significantly higher than the group of Moderately Low SRB in all the dimensions of the SAS-A, obtaining moderate effect sizes for the first dimension (d = 0.51) and low values for the second and third dimensions of the SAS-A (d = 0.33 and 0.45, respectively). On the other hand, there were no significant differences between the groups of High and Moderately High SRB in two of the three dimensions. Regarding family functioning, the Non-SRB group scored significantly higher than the High SRB profile, with a large effect size (d = 0.88). Moderate effect sizes were found for the Non-SRB group, which scored higher than the Moderately High SRB profile (d = 0.69), and for the Moderately Low SRB group, which displayed higher scores than the High SRB profile (d = 0.64). On the other hand, the Non-SRB scored significantly higher than the Moderately Low SRB, whereas this last group scored higher than the Moderately High SRB profile in both cases with a small effect size (d = 0.26 and 0.44, respectively). No significant differences were found between the two groups with high oriented scores in SRB.

## 4. Discussion

Fighting school attendance problems requires knowing the characteristics that define the students affected, both personally and socially, including those related to the family. Until now, the relationships between SRB and anxiety disorders and the family environment have mostly been studied from a clinical point of view [6,17,18,42,66]. However, the appearance of SRB can also occur in community samples, and if these individuals do not receive attention according to their needs, it may increase the risk of dire consequences for the young refusers’ psychological adjustment.

From an approach based on the early detection of school attendance problems, the present study sought to alleviate these deficiencies and provide new empirical evidence to that effect. Our purpose was to identify SRB profiles in a community sample of Spanish adolescents and to analyze their relationships with social anxiety and family functioning.

Four SRB profiles (Non-SRB, High SRB, Moderately Low SRB, and Moderately High SRB) were identified using LCA. These results revealed new groups of school refusers in comparison with the previous profiles identified by cluster analysis with Spanish children [25,26,27]. These findings do not allow the first hypothesis to be confirmed. However, all these studies coincide when identifying a group with low scores in all dimensions of SRB, named Non-SRB. Such disparities can stem from both age differences (children vs. adolescents) and the statistical analysis applied (K-means clustering vs. latent class analysis). In the present study, LCA was used because it is considered the best method for identifying homogeneous classes of subjects [67], in contrast with most previous investigations that have used cluster analysis [23,25,26,27,28,75]. This is recognized as a new contribution of this study, and more research with LCA is necessary to corroborate these profiles. On the other hand, future research could introduce profiling by combining not only the four dimensions of SRB analyzed but also other variables of psychoeducational interest that enrich the characteristics of the groups.

Another novel output of this study is its identification of three new SRB profiles: High SRB with high scores in the four factors of the SRAS-R and Moderately Low and High SRB groups with relatively low and high scores in all dimensions of the scale, respectively. These new profiles, characterized by high and low scores on both negative and positive reinforcements, are consistent with the Multiple SRB profile characterized by high scores in three dimensions of the SRAS-R with explanatory factors for both positive and negative reinforcements [24,43,44]. In addition, the identification of groups with moderate scores in SRB seems to be coherent, being a community sample in which it might be expected that moderate levels of SRB, but not totally high levels, are manifested. In fact, the numbers of students tied to these profiles are 741 (Moderately Low SRB) and 611 (Moderately High SRB), which is the 73.4% of the sample size.

Regarding the second aim, the mean scores of the four groups reveal statistically significant differences in all the dimensions of social anxiety. The groups with High SRB and Moderately High SRB were those that showed higher scores in all dimensions of the SAS-A. Considering these profiles as a group with high scores in positive and negative reinforcements, in line with the SRB group by multiple reinforcements, these results support the second hypothesis. The High SRB profile showed higher levels of fear of the negative evaluation of their peers and greater anxiety and avoidance behaviors both in general and in new social situations compared to the rest of the groups. Therefore, this group showed the most maladaptive profile in terms of average scores of social anxiety. In addition, the most significant differences were found between the adolescents who show High SRB and those whose SRB scores were low or moderately low, with large effect sizes. In contrast, the differences between the High SRB and Moderately High SRB groups were statistically non-significant and exhibited a small effect size only in the second factor of the SAS-S (social avoidance and distress in new situations).

These results allowed us to conclude similar ideas to previous studies [21,24,25,26,42,43,44] which have indicated that high scores in SRB based on positive and negative reinforcements are more related to disorders of an internalizing type, such as social anxiety.

As a result of the findings of this study, we can point out that when the adolescent refuses to attend an educational center and this behavior is produced by both negative and positive reinforcement, social anxiety situations are more likely to occur. In addition, according to our data, it seems that social anxiety is experienced both at a more subjective level (fear of negative evaluation of their peers) and at a more objective level (inhibition or avoidance of social situations in both familiar and novel contexts). These facts are of special relevance in this stage of development because they are critical ages for socialization and the development of relationships and interpersonal skills [38,39,40,76]. In this context of development, adolescents learn social skills, build their identity and self-esteem, try problem solving, etc., which are aspects that influence their psychological and social adjustment and academic achievement. However, the correlational nature of these findings means that they must be interpreted with caution. Other variables, such as both internalizing (e.g., generalized anxiety or depression) and externalizing (e.g., aggressiveness) characteristics, not considered in this study, may be contributing to this behavior.

With regard to the family functioning results, no previous studies have examined whether low Family APGAR ratings (which mean higher perceived family dysfunction) correlate with school attendance problems. Regarding the link between family functioning and SRB profiles, individuals assigned to the High SRB group reported lower mean scores for the perception of satisfactory family functioning, thereby supporting the third hypothesis. This leads to them experiencing feelings of low family support in the domains of adaptation, partnership, affection, growth, and resolve. In contrast, the Non-SRB profile reported the highest mean score in family functioning, becoming the group with more adaptive characteristics that will favor adequate development and adjustment during this evolutionary stage. If adolescence is already considered a stage that can lead to changes and tensions, it becomes a particularly difficult and adverse time when young people reside in family contexts with problems for proper functioning due to insufficient resources and support or a psychosocial risk situation. The results of this research support previous findings which warn that an unfavorable family environment is a risk factor for school refusal behaviors [53,54,55,65,77]. Specific family and community involvement activities need to be promoted by schools and centers for absentee students. For example, empirical evidence has revealed that parents with democratic-style family functioning, characterized by affection, control, and the demands of maturity, have adolescents with better emotional and behavioral adjustment [55]. In addition, it has been proven that family practices can significantly decrease chronic absenteeism [76]. To do this, communicating with families about the relevance of regularly attending school; providing techniques to improve communication, conviviality, and familial unity; and establishing parental schools to help families address emotional and educational problems could reduce students’ absenteeism. It is essential that adolescents can actively participate with adults in the different issues that affect them, but always from negotiated conflict resolution and acquiring democratic habits for the development of norms.

Finally, it should be noted that this work has a number of limitations that future research should try to resolve. First, the findings cannot be generalized to other cultures or age groups different from the reference population of the study. Given the scarcity of studies along this line, it is necessary to carry out new studies that analyze the relationships between these three variables to check whether these results coincide with those obtained in samples from other age groups (e.g., children vs. adolescents) and other cultural and socioeconomic contexts (e.g., ethnic minorities or social groups with low economic resources). In addition, although the family APGAR Scale [49] is an easy-to-administer instrument with satisfactory psychometric properties reported from different countries (e.g., Spain [72], China [78], U.S. [79], Colombia [80], and Peru [81]), only five items compose this measure. Thus, future research should assess family functioning with other measures [57,60] and consider the effects of other family practices, such as parenting styles, since findings suggest the important influence of parent–child relationships on adolescents’ psychosocial adjustment [82,83,84]. Some instruments that contemplate parenting styles and could be implemented in future studies are the Parental Socialization Scale (ESPA29) [85] or the Warmth/Affection Scale and Parental Control Scale, both integrated in the Parental Acceptance–Rejection/Control Questionnaire [86].

Second, given the cross-sectional nature of our study and the correlational method used, it is not possible to establish conclusions of causality (cause-and-effect relationships) between the three variables studied, an aspect that should be addressed through prospective longitudinal studies and the use of structural equation models. Furthermore, future studies are encouraged to explore the relationship between school refusal behavior profiles and other variables, both social (e.g., peer relationships, socioeconomic context) and individual (e.g., aggressiveness, self-concept). Finally, another of the limitations of our study is that only self-report measures were used by the adolescents. It would be advisable for future studies to adopt an evaluation perspective with multiple informants (parents and teachers), in which other evaluation instruments such as diagnostic interviews, parent/teacher control lists, and school attendance records would be incorporated, as King et al. [87] proposed.

Despite these limitations, this study provides some novel information for understanding the complex heterogeneity of cases of school refusal behavior. For the first time, school refusal behavior profiles were identified in Spanish adolescents by applying one of the more supported methods to identify profiles today, latent class analysis, instead of cluster analysis. On the other hand, this work confirmed that multiple factors (both positive and negative reinforcements) may be maintaining school attendance problems in the youth population. Special attention to these groups (the High SRB and Moderately High SRB profiles) should be paid from the manifestation of the first symptoms (e.g., continued delays in class access, lack of interest in studies, substance use, poor family participation in school, etc.) because these groups achieved the highest mean scores for social anxiety problems and worse perception of family functioning. Supporting previous research, these groups characterized by multiple reinforcements are the most maladaptive profiles and need special attention on a preventive basis. Another important contribution of this study is the confirmation of the fact that school attendance problems affect not only clinical samples but also populations of students who regularly attend school. Finally, it should be noted that this study has overcome the traditional approach that analyzes school refusal behavior as an individual problem to know how other variables in the social context, such as the perception of family functioning, affect adolescents’ development.

Today, health is understood as the complete state of physical, mental, and social well-being [88], and healthy childhood development implies a child’s regular school attendance. Therefore, educational policies are needed that support health promotion and prevent school absenteeism. In this sense, we can emphasize the necessity of implementing prevention programs focused on developing positive interpersonal relationships between peers and family members and reducing the emotional distress that young people may experience in different school or family situations. In addition, adolescence is considered to be an optimal time to develop skills related to health and general well-being since the interventions will have time to take effect and impact on the improvement of the individual’s health in the coming years [89].

On the other hand, the influence of other variables more related to the motivational factors of learning or family context should not be ignored. A teaching methodology that is not adapted to the needs and interests of students or families that are carefree about the academic career of their children could also be the reason behind school attendance problems. The strengthening of positive relationships among learning community members, vocational counselling, and parents’ schools could prevent these situations. All these actions require that teachers, parents or guardians, and mental health professionals work as a multidisciplinary team to achieve the same goal, which is the regular school attendance of all students [90].

## 5. Conclusions

This study verified the existence of different groups of students with SRB: those with non-school refusal behavior, high school refusal behavior, moderately low school refusal behavior, and moderately high school refusal behavior. The groups identified are novel with respect to previous research, with this being the first study carried out with Spanish adolescents and using latent class analysis. Groups with moderately low and high scores in SRB were the most predominant, representing 72% of the total sample, which may be due to it being a non-clinical sample. The high school refusal behavior profile was the group with the most maladaptive scores in social anxiety and family perception functioning, showing similarities with the mixed school refusal behavior profile identified in previous studies. This implies that groups with high scores of school refusal behavior for positive and negative reinforcement at the same time are the groups at a greater risk of having social anxiety and a worse perception of family functioning. Improving social skills, developing positive interpersonal relationships, and reducing emotional distress can lead to the prevention of school attendance problems and other related emotional problems [91,92].

## Figures and Tables

**Table 1:** Sample distribution across gender and age.

Gender	Age				Total
	15	16	17	18	
Boys	204	299	228	134	865
	11.1%	16.2%	12.4%	7.3%	47%
Girls	260	310	259	148	977
	14.1%	16.8%	14.1%	8%	53%
Total	464	609	487	282	1842
	25.2%	33%	26.5%	15.3%	100%

**Table 2:** Fit indices of the latent class analysis (LCA); values in bold show the best model fit.

Number of Classes	Bayesian Information Criterion (BIC)	Entropy
2 classes	18101.54	0.72
3 classes	17427.31	0.74
4 classes	17199.87	0.75
5 classes	17262.62	0.70

**Table 3:** Means and standard deviations obtained by the four clusters in SAS-A dimensions.

SAS-A ^1^ Dimensions	Non-SRB ^5^		High SRB		Moderately Low SRB		Moderately High SRB		Statistical Significance	
	*M*	*SD*	*M*	*SD*	*M*	*SD*	*M*	*SD*	*F _(3,1839)_*	*η^2^*_p_
FNE ^2^	14.07	5.74	19.59	7.49	16.04	5.33	18.97	6.18	59.34 *	0.09
SAD-New ^3^	12.01	4.78	16.53	5.47	13.78	4.43	15.29	4.71	46.38 *	0.07
SAD-General ^4^	7.55	3.29	10.35	3.97	8.74	3.13	10.21	3.48	48.23 *	0.08
APGAR	14.92	3.16	12.31	2.80	14.15	2.91	12.88	2.89	51.22 *	0.08

^1^ Social Anxiety Scale for Adolescents; ^2^ fear of negative evaluation; ^3^ social avoidance and distress in new situations; ^4^ social avoidance and distress that is experienced more generally in the company of peers; ^5^ school refusal behavior; * *p* < 0.001.

**Table 4:** Cohen’s d value for post hoc contrasts between cluster groups for SAS-A dimensions.

Dimensions SAS-A ^1^	Moderately Low SRB ^5^ vs. Moderately High SRB	Moderately Low SRB vs. High SRB	Moderately Low SRB vs. Non-SRB	Moderately High SRB vs. High SRB	Moderately High SRB vs. Non-SRB	High SRB vs. Non-SRB
FNE ^2^	−0.51	−0.60	0.36	-	0.81	0.82
SAD-N ^3^	−0.33	−0.58	0.39	−0.25	0.69	0.88
SAD-G ^4^	−0.45	−0.48	0.38	-	0.78	0.77
APGAR	0.44	0.64	−0.26	-	−0.69	−0.88

^1^ Social Anxiety Scale for Adolescents; ^2^ fear of negative evaluation; ^3^ social avoidance and distress in new situations; ^4^ social avoidance and distress that is experienced more generally in the company of peers; ^5^ school refusal behavior.
